# Clustering of Unhealthy Diets, Inflammation Levels, and Depressive Symptoms in Chinese Adolescents: A Longitudinal Study

**DOI:** 10.3390/nu18162636

**Published:** 2026-08-12

**Authors:** Honglv Xu, Dongyue Hu, Xiaoxiao Li, Jiaxing Yang, Danyun Rui, Na Liu, Huijuan Wu, Jieru Yang, Litao Chang, Feng Jiao

**Affiliations:** 1 School of medicine, Kunming University, Kunming 650214, China; 2 Division of School Health, Yunnan Provincial Center for Disease Control and Prevention, Kunming 650500, China; 3 School of Public Health, Kunming Medical University, Kunming 650500, China

**Keywords:** adolescent, depressive symptoms, unhealthy diets, inflammatory, longitudinal study

## Abstract

Background/Objectives: Studies suggest a potential link between dietary behaviors, inflammation, and depressive symptoms in adolescents, but longitudinal evidence remains scarce. This study examines the longitudinal associations among unhealthy diets, inflammatory markers, and depressive symptoms in Chinese adolescents. Methods: This prospective longitudinal study enrolled 1693 middle school students (52.3% female) from Yunnan Province, China. Participants were assessed at five waves: October 2022 (T1), June 2023 (T2), October 2023 (T3), June 2024 (T4), and October 2024 (T5). Unhealthy dietary behaviors were assessed using a Food Frequency Questionnaire, and depressive symptoms were measured with the Children’s Depression Inventory. Blood samples were collected to measure inflammatory markers. Generalized estimating equations, generalized linear models, and path analysis were applied to examine the associations and mediating pathways. Results: Prevalence of depressive symptoms increased from 26.3% to 37.3% over 2 years. Compared with Q1 (the lowest quartile), Q4 of unhealthy dietary scores was significantly associated with an increased risk of positive depression symptoms (adjusted *OR* = 1.20). IL-4 (*β* =−0.26–−0.17) and CRP (*β* = 0.64–1.07) were associated with depressive symptoms. However, the mediating role of inflammation in the association between unhealthy dietary scores and depressive symptoms was not statistically significant. Conclusions: Chinese adolescents exhibit both a high clustering of unhealthy dietary behaviors and notably elevated levels of inflammation. Our longitudinal study indicates that the clustering of unhealthy diets and inflammation levels are associated with depressive symptoms among Chinese adolescents.

## 1. Introduction

Adolescent depression is not only a major public health challenge globally but also the leading cause of disability and the most significant contributor to the burden of disease in this age group [1].Globally, the prevalence of depressive symptoms among adolescents is on the rise [2,3,4], and the same trend is observed among Chinese adolescents [5]. A systematic review and meta-analysis encompassing 96 studies across 29 countries worldwide showed that from 1989 to 2022, the pooled prevalence rates of mild-to-severe, moderate-to-severe, and major depression among children and adolescents globally were 21.3%, 18.9%, and 3.7%, respectively [1]. Another systematic review and meta-analysis showed that from 2001 to 2020, the global point prevalence of elevated self-reported depressive symptoms among adolescents was 34%, the point prevalence of elevated depressive symptoms at a high level increased from 24% between 2001 and 2010 to 37% between 2011 and 2020 [3]. Recent studies indicate that the prevalence of depressive symptoms among adolescents is pronounced. A survey covering 134 secondary schools across Australia revealed that the prevalence of depressive symptoms among eighth-grade students was 58.6%, of which 43.5% exhibited mild-to-moderate depressive symptoms and 15.1% presented with clinical-level depressive symptoms [6]. The prevalence of depressive symptoms among Chinese adolescents is 28.3%, with a higher rate in females (30.9%) than in males (25.5%) [7]. Overall, adolescent depression presents distinct characteristics across different developmental stages [8]. A cohort study from Australia reported that the estimated mean age of first reported depressive symptoms in adolescents was 14.1 years, the cumulative incidence of developing any clinical-level depressive symptoms during adolescence was 65%, and 54% of depressive symptoms followed a chronic course between the ages of 10 and 18 years [9]. Depressive symptoms in adolescents are predominantly mild to moderate in severity. For instance, 66.8% of adolescents in the United States present with persistent mild depressive symptoms, while 19.1% exhibit persistent moderate symptoms [8]. In China, mild depressive symptoms account for 85% of cases [10]. Furthermore, depressive symptoms in adolescents not only affect memory, attention, and executive function during adolescence but also influence depressive symptoms in adulthood [11,12]. The Québec Longitudinal Study of Child Development in Canada indicates that depressive symptoms during adolescence are associated with higher levels of depressive symptoms in early adulthood (*β* = 1.08) and are linked to receiving less social support [13].

Unhealthy dietary behaviors characterized by skipping breakfast, irregular mealtimes, nighttime eating, and frequent consumption of snacks, sugar-sweetened beverages (SSBs), and ultra-processed foods (UPFs) are highly prevalent among adolescents worldwide. A survey covering children and adolescents aged 6–17 years from five Mediterranean countries revealed that 95% of participants consumed an unhealthy diet high in energy, fat, and sugar daily, with a median intake of 1.8 servings per day [14]. In Colombia, UPFs account for 17.6% of total dietary energy intake among adolescents, with a higher proportion observed in urban adolescents (19.0%) and those from middle socioeconomic strata (23.0%) [15]. Adolescents in Romania exhibit addictive tendencies toward unhealthy diets, with reported consumption rates of 38.9% for fried potatoes, 37.05% for hamburgers, 31.65% for shawarma, and 30.08% for snacks [16]. A survey in Iraq showed that 32.68% of adolescents skip breakfast, 53.57% consume snacks outside of three main meals, 39.82% consume fast food more than three times per week, and 61.07% consume SSBs one to three times per day [17]. Additionally, a study in Brazil showed that over the past decade, diet quality among adolescents aged 15–19 years has deteriorated, with a decrease in healthy food intake and an increase in unhealthy food intake. Specifically, daily consumption of fruits and dairy products decreased by 20 g and 59 g, respectively, among girls; among boys, daily dairy intake decreased by 55 g, while fast food intake increased by 22 g per day [18].

Limited research suggests that unhealthy dietary behaviors are also highly prevalent among Chinese adolescents. In eastern China, the reported rate of adhering to daily breakfast consumption among adolescents is 71.0% [19]. In western China, the reported rate of adhering to daily breakfast consumption is 51.1%, while the rate of never consuming late-night snacks is 28.7% [20]. Our preliminary survey indicates that the reported consumption rates among Chinese adolescents for processed meat, processed eggs, preserved fruits, instant foods, salty snacks, sweet snacks, and SSBs range from 45.6% to 89.5% on a weekly basis. The consumption rates among males range from 46.4% to 89.1%, while those among females range from 44.8% to 89.9% [7]. We also found that the reported consumption rates of Western-style fast food, Chinese-style fast food, and takeaway fast food among adolescents were 28.0%, 34.2%, and 19.6%, respectively. The consumption rates among males were 28.4%, 33.1%, and 19.5%, respectively, while those among females were 27.5%, 35.2%, and 19.7%, respectively [21].

Adolescent unhealthy dietary behaviors are influenced by a variety of factors, including personal factors (e.g., smartphone addiction, screen time, nighttime eating, watching mukbang, negative self-perceived health status, obesity, body image, etc.), family factors (e.g., parental education level, parental age, family socioeconomic status, etc.), environmental factors (e.g., accessibility of unhealthy food at school and its surrounding areas, area of residence, level of urbanization, etc.), and social factors (e.g., food and beverage advertising on digital media, unhealthy food advertising, food marketing targeting adolescents, peer relationships, etc.) [14,15,22,23,24,25,26,27,28,29].

Current epidemiological evidence indicates that unhealthy dietary behaviors and dietary patterns are associated with depressive symptoms in adolescents [30,31,32,33]. A meta-analysis shows that healthy dietary patterns are negatively associated with the risk of depressive symptoms in children and adolescents [34]. The National Survey of Children’s Health in the United States indicates that unhealthy dietary habits play a partial mediating role in the association between living conditions and the risk of depression [35]. Multiple studies have demonstrated across various dimensions the association between dietary behaviors and depressive symptoms among Chinese adolescents, indicating that unhealthy dietary behaviors are positively associated with depressive symptoms [19], while dietary quality is negatively associated with depressive symptoms [36]. For instance, a study conducted in eastern China found that compared with students who adhered to daily breakfast consumption, those who ate breakfast six days a week, four to five days a week, and three or fewer days a week had odds ratios (*OR*) for depressive symptoms of 1.32, 1.66, and 1.74, respectively [19]. Additionally, skipping breakfast (*OR* = 2.56) was independently associated with depressive symptoms among adolescents aged 11 to 19 years [37]. In addition to breakfast, night eating symptoms are also associated with depressive symptoms in adolescents [38]. In western China, the number of days of breakfast intake (*β* = −0.71 for males, *β* = −0.77 for females) and the number of days of late-night snack intake (*β* = 0.39 for males, *β* = 0.17 for females) were associated with depressive symptom scores among adolescents [20]. Moreover, studies have reported that higher sugar intake is associated with depressive symptoms in adolescents aged 18 to 19 years [39]. Our previous study also found that the consumption of processed foods (*β* = 0.15–0.29), carbonated beverages (*β* = 0.15–0.29), and energy beverages (*β* = 0.21–0.22) was associated with depressive symptoms in adolescents [7]. Conversely, healthy dietary behaviors may exert a protective effect against the development of depressive symptoms in adolescents. For instance, a study in Australia found that adolescents with higher total dietary fiber intake had a lower prevalence of depressive symptoms [40]. A systematic review further indicated that the intake of magnesium, vitamin B_12_, dietary fiber, fruits, vegetables, and fish was negatively associated with depressive symptoms in adolescents [41].

Inflammation serves as a biological process that mediates the interplay between organisms and their environment [42], with holistic dietary patterns playing a critical role in modulating inflammatory responses in adolescents [43]. Diet may influence mental health through multiple pathways, among which modulating inflammatory responses is a key pathway [44]. Limited research indicates that diet is associated with inflammatory levels, with pro-inflammatory diets potentially elevating systemic inflammation levels, whereas anti-inflammatory diets may help reduce systemic inflammation levels [45,46]. A systematic review demonstrates that high-quality diets rich in vegetables, fruits, whole grains, fiber, and healthy fats can improve low-grade inflammation in children and adolescents [47]. Adherence to such healthy dietary patterns and their components is associated with reduced levels of inflammatory biomarkers, including C-reactive protein (CRP), interleukin-6 (IL-6), and tumor necrosis factor-alpha (TNF-α), in children and adolescents [48]. Conversely, unhealthy pro-inflammatory dietary patterns increase inflammatory levels. Such dietary patterns are typically rich in refined starches, sugar, saturated fatty acids, and trans fatty acids, yet deficient in natural antioxidants, fiber, and omega-3 fatty acids. They may activate the innate immune system by promoting the production of pro-inflammatory cytokines and reducing the generation of anti-inflammatory cytokines [49]. For instance, studies have found that unhealthy diets may elevate levels of inflammatory cytokines such as IL-1β, IL-4, IL-6, IL-10, TNF-α, and CRP [46]. Saturated fatty acids are positively correlated with soluble intercellular adhesion molecule-1, IL-6, and high-sensitivity CRP [50]. Furthermore, a study conducted in Australia demonstrated that high intake of red meat, takeaway foods, processed foods, and sweets was positively associated with serum CRP levels in 17-year-old adolescents [51].

Limited research suggests an association between immunometabolic function and inflammatory levels with depression [52]. Patients with major depressive disorder exhibit elevated levels of peripheral inflammatory markers [53], such as higher CRP levels (*OR* = 1.12) [54]. CRP has been established as an independent predictor of adolescent depression [55]. Compared with non-depressed individuals, patients with depression have a higher CRP to albumin ratio and elevated CRP levels [56]. Meanwhile, IL-6 levels are also associated with the risk of adolescent depression. A longitudinal study indicated that elevated IL-6 levels in children at age 9 were associated with an increased risk of depressive symptoms by age 18 (*OR* = 1.55) [57]. A birth cohort study showed that higher IL-6 levels during the second trimester were associated with more severe depressive symptoms in offspring during adolescence [58]. Additionally, a systematic review and meta-analysis showed that lower dietary inflammation was associated with a reduced risk of developing depressive symptoms (*RR* = 0.76) [59]. In summary, diet is associated with inflammatory levels, and inflammation serves as a predictor of depressive symptoms. Therefore, inflammation may play a mediating role in the association between diet and depressive symptoms, and interventions targeting dietary behaviors to reduce inflammatory levels may represent an effective strategy for improving depressive symptoms in adolescents [60].

Although several studies have explored the associations among dietary behaviors, inflammatory levels, and depressive symptoms in adolescents, research focusing on multi-ethnic adolescents in China remains very limited. Moreover, existing findings are inconsistent. For instance, some studies have reported insufficient evidence for associations between the intake of vitamins B6, C, D, and E, iron, copper, zinc, omega-3 fatty acids, carbohydrates, and dietary fat and depressive symptoms [41]. The Prospective Community-Based Cohort Study in the United Kingdom showed that the association between diet quality and depressive symptoms during adolescence was not significant [61]. Furthermore, the mechanistic role of inflammatory levels in the association between unhealthy dietary behaviors and depressive symptoms in adolescents remains unclear. Yunnan Province, located in southwestern China and bordering Myanmar, Laos, and Vietnam, is home to the largest number of ethnic minority groups in the country. Influenced by diverse dietary cultures, ethnic eating habits, and relatively underdeveloped economic conditions, the dietary behaviors of adolescents in Yunnan exhibit distinct characteristics. This study employs a longitudinal design to investigate the longitudinal association between unhealthy dietary behaviors and depressive symptoms among multi-ethnic adolescents in Yunnan Province, China, as well as the mediating role of inflammatory levels in this association.

## 2. Materials and Methods

### 2.1. Study Population

**Figure 1:**
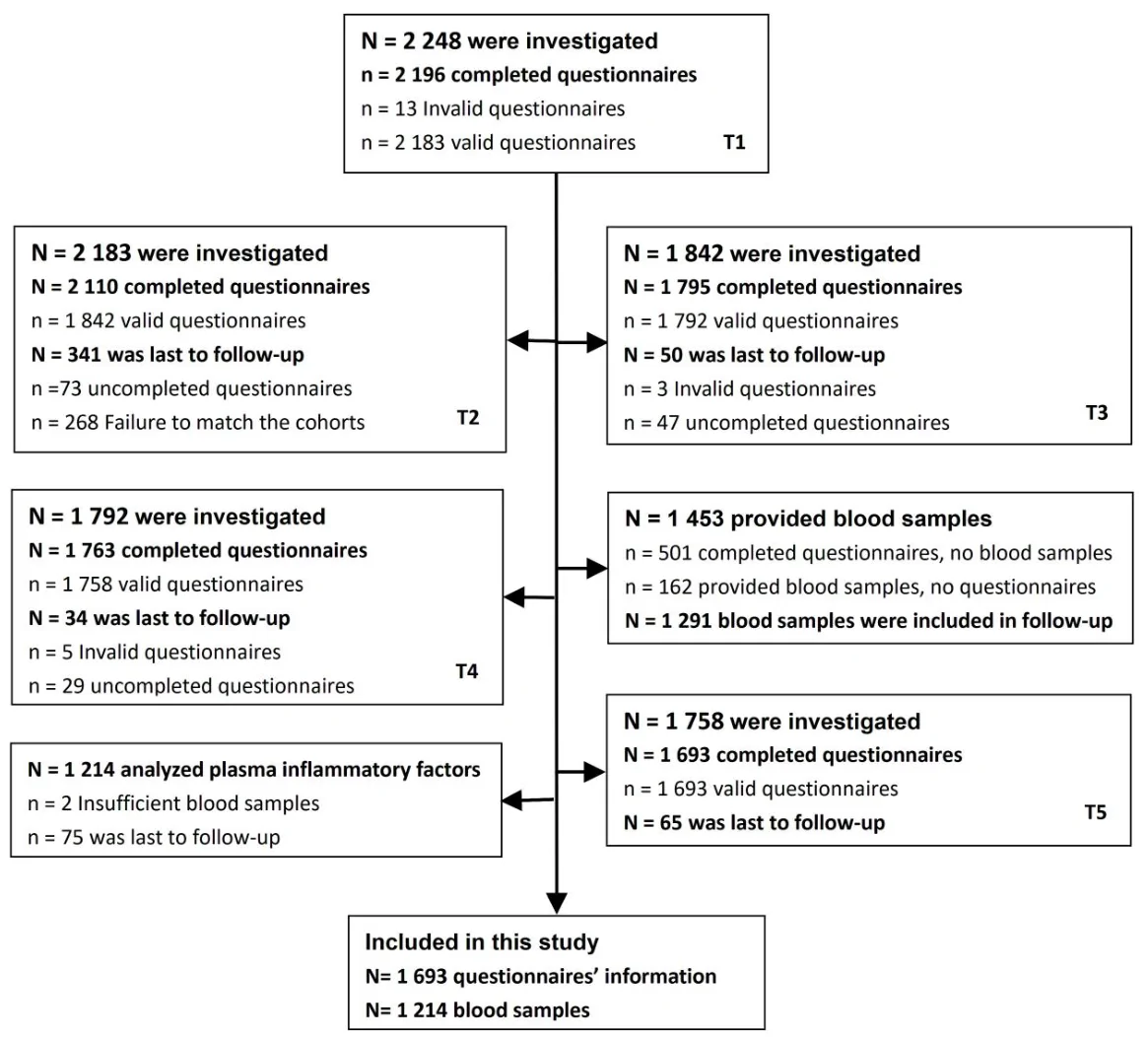
Flow chart of participants.

This prospective longitudinal study enrolled middle school students from four schools across three regions of Yunnan Province, China. The baseline survey (T1) was conducted in October 2022. Follow-up surveys were then carried out at the following time points: June 2023 (T2), October 2023 (T3), June 2024 (T4), and October 2024 (T5), resulting in a total follow-up duration of two years. At T1, 2248 adolescents were included in the investigation, and 2183 provided valid questionnaires. Based on the valid sample at T1, 2183 individuals were followed up at T2, with 1842 valid questionnaires completed. At T3, 1842 individuals were surveyed, and 1792 valid questionnaires were collected. A total of 1453 individuals provided blood samples, and after screening, 1291 blood samples were included in the subsequent follow-up. At T4, the investigation continued for 1792 individuals, with 1758 valid questionnaires collected, and this group was included in the follow-up at T5, ultimately obtaining 1693 valid questionnaires. Figure 1 shows the Flow chart of participants. The attrition rates for follow-up was 1.9% to 15.6%. Therefore, 1693 questionnaires were included in the cohort analysis. After excluding insufficient samples and lost to follow-up samples, 1214 blood samples were included in analysis. Of these, 808 (47.7%) were male and 885 (52.3%) were female. In terms of ethnicity, the participants identified as Han (37.2%, *n* = 629), Yi (37.1%, *n* = 628), Lahu (8.4%, *n* = 143), Hani (3.9%, *n* = 66), Dai (4.6%, *n* = 78), and other ethnic groups (8.8%, *n* = 149). Regarding residence, 1278 (75.5%) resided in rural areas, while 415 (24.5%) resided in urban areas. The distribution of the remaining demographic variables has been described in our previous work [20]. Table 1 presents the distribution of demographic variables in relation to the prevalence of depressive symptoms. This study was conducted in accordance with the World Medical Association’s Declaration of Helsinki. The protocol was approved by the participating schools, and informed consent was obtained from all adolescent participants and their parents. Participation was anonymous and voluntary, with the right to withdraw at any time during the follow-up. All data were kept strictly confidential and used solely for the purposes of this analysis. The study was approved by the Medical Ethics Committee of the School of Medicine, Kunming University (Approval Number: 20210222).

### 2.2. Survey Methods

Using a random cluster sampling method, this cross-sectional questionnaire survey was conducted among middle school students in 11 counties of Yunnan Province. Full methodological details are available in our previously published work [21]. From this cross-sectional cohort, four middle schools were selected for the longitudinal follow-up study based on the criteria of high multi-ethnic representation and strong participant compliance. All enrolled seventh-grade students from the selected schools were included in the study and completed a self-administered questionnaire in a classroom setting. The survey collected data on demographic variables, unhealthy dietary habits, depressive symptoms, and potential confounding factors for adolescent depression, including self-perceived academic stress, self-perceived socioeconomic status, family changes, hospital experience, smoking, alcohol consumption, family history of depression, left-behind experience, physical activity, the impact of COVID-19, symptoms of insomnia, and screen time. The survey was administered in person by trained staff, who provided face-to-face assistance and ensured that all adolescents completed the questionnaires independently without discussion. The process took approximately 15–20 min. Following collection, all questionnaires were reviewed for completeness, and any omissions were addressed promptly by the surveyors.

### 2.3. Demographic Variables

The demographic variables investigated include sex, age, ethnicity, the only child in the family, family type, residence, self-perceived socioeconomic status, the number of close friends, parental education levels, and parental occupations.

### 2.4. Evaluation of Depressive Symptoms

Depressive symptoms were measured employing the validated Chinese version of the Children’s Depression Inventory (CDI) [62,63]. The CDI is a widely used self-report scale that evaluates depressive symptoms in individuals aged 7–17, based on their experiences during the preceding two weeks. The CDI is composed of 27 items, grouped into five dimensions: anhedonia, negative mood, negative self-esteem, ineffectiveness, and interpersonal problems. Each item is rated on a 3-point scale (“occasionally,” “often” and “always”), with scores ranging from 0 to 2 per item, resulting in a total possible score of 0–54. Higher scores represent more severe depressive symptoms. A total score of ≥19 was defined as indicative of depressive symptoms, while a score below this threshold was classified as negative. Previous studies involving Chinese children and adolescents have confirmed the good reliability and validity of the CDI, supported by a Cronbach’s α of 0.84–0.85 [64,65]. In the present study, the Cronbach’s α across all five time points (T1 to T5) ranged from 0.86 to 0.90.

### 2.5. Unhealthy Diets Evaluation

Unhealthy diets were assessed with a food frequency questionnaire developed by our research team [21,66]. This study investigated 22 types of unhealthy dietary consumption, including Western fast food (e.g., hamburgers, fried chicken), Chinese fast food (e.g., Shaxian Snacks, street snacks), fast food delivery (e.g., Ele.me, Meituan), cured meats (e.g., Xuanwei Ham, cured pork ribs), pickled vegetables (e.g., preserved Vegetable, dried Pickled Radish), and barbecued food (e.g., grilled meat skewers, grilled noodles), staple processed food (e.g., fried bread stick, fried dough cake), processed meat (e.g., Chinese bacon, canned meat), processed eggs (e.g., salted duck egg, spiced corned egg), preserved fruits (e.g., preserved apple, Winter melon preserves), puffed food (e.g., potato chips, prawn Crackers), Ultra-processed foods containing emulsifiers (e.g., cream cakes, ice cream), instant foods (e.g., instant noodles, self-heating rice), salted snacks (e.g., spicy strip, salty crackers), sugar (e.g., candies, granulated sugar), sweet snacks (e.g., chocolate, sachima), carbonated beverages (e.g., Coca Cola, Sprite), juice beverages (e.g., orange juice, apple juice), tea beverages (e.g., Iced black tea, bottled green tea), energy beverages (e.g., Red Bull, Luhu), milk beverages (e.g., milk tea, wang zai milk), and other SSB (e.g., vitamin beverages, sports beverages). We assessed the consumption frequency of unhealthy diets with concise questions. As an example, adolescents were asked, “Over the past month, how many bottles of Sprite (500 mL per serving) did you typically drink each week?” Responses were recorded on a predefined scale with 10 options: 0, 1, 2, 3, 4, 5, 6, 7, 8, or ≥9 times, which they selected according to their actual consumption.

### 2.6. Analysis of Inflammatory Factor Levels

Plasma levels of IL-4 and CRP were measured using double-antibody sandwich enzyme-linked immunosorbent assay (ELISA) kits (Ruixin Biotechnology Co., Ltd., Quanzhou, China). The main detection steps were as follows: ① Standards, blanks, and plasma samples were added to 96-well plates. ② Detection antibodies labeled with horseradish peroxidase (HRP) were added. ③ After incubation for 1 h and five repetitions of plate washing, a chromogenic substrate was added. Following a 15-min incubation, the reaction was terminated using a stop solution. ④ Absorbance was read at a wavelength of 450 nm using a microplate reader (TECAN Infinite F50, Tecan Austria GmbH, Grödig, Austria). ⑤ A quadratic regression curve was used to fit the standard curve (all curves *R*^2^ > 0.99), and the concentrations of IL-4 and CRP in the samples were calculated. Quality control was strictly implemented throughout the experiment, with high and low controls set for each plate. The measured values of the quality control samples were considered to be under control when they fell within the accuracy range provided by the manufacturer. The inter-plate coefficient of variation (CV) was calculated using the formula CV = [standard deviation (*σ*)/mean (*μ*)] × 100%. The inter-plate CVs for high and low IL-4 controls were 5.43% and 13.92%, respectively; for CRP, they were 10.37% and 10.27%. All inter-plate CVs were less than 15%, meeting the requirements of the kit.

### 2.7. Statistical Analysis

Data management using EpiData 3.0. After data verification, statistical analyses were conducted using SPSS (version 23.0; SPSS Inc., Chicago, IL, USA) and Mplus (version 7.4). The statistical analyses comprised descriptive statistics, *χ*^2^ tests, generalized estimation equation (GEE), the generalized linear model (GLM), path analysis (PA), and structural equation model (SEM). First, a *χ^2^* test was used to compare differences in the prevalence rates of depressive symptoms among adolescents with different demographic characteristics. Second, we employed the GEEs to examine the associations of unhealthy diets with depressive symptoms, conducting stratified analyses by sex. The baseline unhealthy dietary behavior scores were categorized into quartiles (Q1–Q4), with Q1 serving as the reference group. A binary dependent variable was defined as a depression symptom score ≥19, indicating positive depression symptoms, and logistic regression models based on GEE were applied for analysis. Third, we utilized the GLMs to analyze the associations of inflammation with depressive symptoms, conducting stratified analyses by sex. In the GEEs and GLMs, unhealthy diet scores and inflammatory factor levels were treated as independent variables, while depressive symptom scores were the dependent variable. We established two models: an unadjusted model (Model 1) and a model adjusted for demographic variables and potential influencing factors of depressive symptoms (Model 2). Fourth, We performed a path analysis (PA) to investigate the longitudinal pathways among unhealthy diets (UD), inflammation, and depressive symptoms (DS) over time-point (T). In PA, the unhealthy diet scores at T3 is specified as an exogenous variable, whereas IL-4, CRP, and depressive symptom scores at T3, along with unhealthy diet scores and depressive symptom scores at T4 and T5, are modeled as endogenous variables. The path model was specified to include three types of effects: (a) autoregressive effects (T3 DS → T4 DS → T5 DS; T3 UD → T4 UD → T5 UD), (b) cross-lagged effects (T3 UD → T4 DS; T3 UD → T5 DS; T4 UD → T5 DS), and (c) contemporaneous cross-sectional effects (T3 UD → T3 DS; T3 UD → T3 IL-4; T3 UD → T3 CRP). The model fit indices indicated that the path model fit the data well, as shown in the following values: AIC = 56,450.888, BIC = 56,629.447, Sample-Size Adjusted BIC = 56,518.272, Chi-Square Test of Model Fit = 5569.736 (*p* < 0.01), RMSEA = 0.022, CFI = 0.999, TLI = 0.997, SRMR = 0.019. Furthermore, a PA was applied to test the mediating role of inflammatory in the association between unhealthy diets and depressive symptoms in adolescents. Two separate mediation models were conducted to examine the mediating roles of IL-4 and CRP at T3 in the relationship between T3 unhealthy diet scores and depressive symptoms at T3, T4, and T5, with the independent variable (unhealthy diet scores) and dependent variable (depressive symptom scores). In Model 1, IL-4 served as the mediator; in Model 2, CRP served as the mediator. The path analysis was conducted using maximum likelihood (ML) estimation, and the significance of the indirect effect was tested via bias-corrected bootstrap [67]. The statistical significance level was set at *α* = 0.05.

## 3. Results

### 3.1. Depressive Symptoms Across Sociodemographic Groups in Adolescents

The prevalence of depressive symptoms showed a clear upward trend from T1 to T5 across all groups. The rates were 26.3%, 31.3%, 34.0%, 36.0%, and 37.3% for the total sample; 21.7%, 25.1%, 28.7%, 32.2%, and 31.6% for males; 30.5%, 36.9%, 38.9%, 39.4%, and 42.5% for females. Table 1 presents the prevalence of depressive symptoms among adolescents with different demographic characteristics. The prevalence of depressive symptoms varied significantly for males based on age, ethnicity, family residence, number of close friends, self-rated family economic status, mother’s occupation (*p* < 0.05). Also, statistically significant differences in the prevalence of depressive symptoms were observed among females for all demographic variables except for family residence and the only child in the family (*p* > 0.05).

### 3.2. Unhealthy Diets and Depressive Symptoms

The prevalence of reported consumption of western fast food, Chinese fast food, fast food delivery, cured meats, pickled vegetables, barbecued food, staple processed food, processed meat, processed eggs, preserved fruits, puffed food, Ultra-processed foods containing emulsifiers, instant foods, salted snacks, sugar, sweet snacks, carbonated beverages, juice beverages, tea beverages, energy beverages, milk beverages, and other SSB were 38.2%, 40.6%, 24.7%, 56.6%, 59.6%, 56.7%, 61.1%, 68.0%, 45.9%, 53.4%, 79.5%, 78.0%, 60.0%, 71.5%, 67.1%, 64.8%, 67.2%, 72.9%, 71.6%, 47.8%, 75.2%, and 51.6%, respectively. Table 2 presents the association of unhealthy diets score with depressive symptoms among Chinese adolescents. After adjusting for confounding variables (model 2), compared with the Q1 group, the Q4 group was significantly associated with an increased risk of positive depression symptoms (adjusted *OR* = 1.20, 95%*CI*: 1.02–1.41) among adolescents. Moreover, the linear trend test across the four groups reached statistical significance (*P* for trend = 0.013), indicating a clear dose-response increasing relationship between the level of unhealthy dietary behavior exposure and the risk of positive depression symptoms. Sex-stratified analysis showed the Q4 group was significantly associated with an increased risk of positive depression symptoms (adjusted *OR* = 1.25, 95%*CI*: 1.01–1.55) among females.

### 3.3. Inflammatory Level and Depressive Symptoms

Inflammatory markers were measured only at T3. The levels of IL-4 and CRP were (median, inter-quartile range, 13.012, 6.662) pg/mL, and (3.396, 1.711) mg/mL, respectively. After adjusting for confounding variables, their associations with depressive symptoms at T3, T4, and T5 were as follows. At T3, IL-4 (*β* = −0.17, 95*%CI*: −0.32–−0.02, *p* = 0.023) and CRP (*β* = 0.64, 95*%CI*: 0.05–1.24, *p* = 0.035) were associated with depressive symptoms in the total sample, and IL-4 (*β* = −0.22, 95*%CI*: −0.43–−0.02, *p* = 0.034) was associated with depressive symptoms in females. At T4, IL-4 (*β* = −0.26, 95*%CI*: −0.42–−0.09, *p* = 0.002) and CRP (*β* = 0.87, 95*%CI*: 0.21–1.53, *p* = 0.010) were associated with depressive symptoms in the total sample, and IL-4 (*β* = −0.25, 95*%CI*: −0.48–−0.02, *p* = 0.033) was associated with depressive symptoms in females. At T5, IL-4 (*β* = −0.26, 95*%CI*: −0.44–−0.09, *p* = 0.004) and CRP (*β* = 1.07, 95*%CI*: 0.37–1.78, *p* = 0.003) were associated with depressive symptoms in the total sample, and both IL-4 (*β* = −0.30, 95*%CI*: −0.56–−0.05, *p* = 0.021) and CRP (*β* = 1.18, 95*%CI*: 0.21–2.14, *p* = 0.017) were associated with depressive symptoms in males. Table 3 shows the association of inflammatory level with depressive symptoms among Chinese adolescents.

### 3.4. Association Path Between Unhealthy Diets, Inflammatory Levels, and Depressive Symptoms in Adolescents

**Figure 2:**
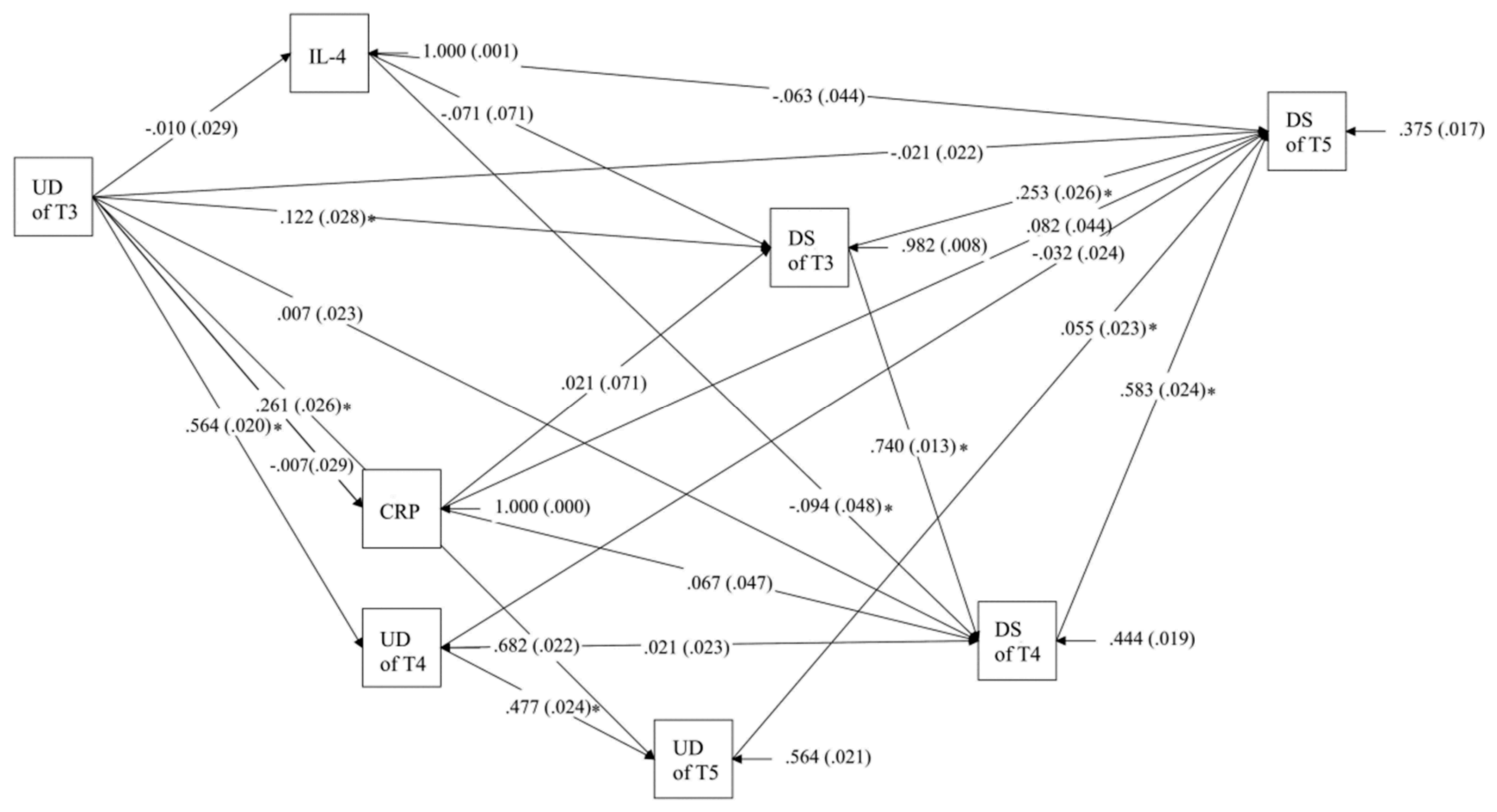
Association path between unhealthy diets, inflammatory levels, and depressive symptoms among Chinese adolescents. * *p* < 0.05.

Path analysis revealed substantial temporal stability in both unhealthy diet scores and depressive symptoms over the follow-up period. Specifically, unhealthy diet scores showed strong tracking effects from T3 to T4 (*β* = 0.564) and from T4 to T5 (*β* = 0.477), indicating that adolescents with poorer dietary habits at one time point tended to maintain these patterns over time. Similarly, depressive symptoms exhibited high stability across waves, with particularly strong continuity from T3 to T4 (*β* = 0.740) and T4 to T5 (*β* = 0.583), suggesting that depressive symptoms once established tend to persist. Unhealthy diet scores were concurrently associated with depressive symptoms at the same time points, specifically at T3 (*β* = 0.122) and T5 (*β* = 0.055). Notably, among the inflammatory markers, only IL-4 at T3 showed a modest but statistically significant negative association with depressive symptoms at T4 (*β* = −0.094), suggesting that higher IL-4 levels might exert a slight protective or compensatory effect on subsequent mood regulation, although this effect was relatively small in magnitude. Table 4 and Figure 2 presents the association path between unhealthy diets, inflammatory levels, and depressive symptoms among Chinese adolescents.

### 3.5. Mediating Role of Inflammatory Levels in the Association Between Unhealthy Diets and Depressive Symptoms in Adolescents

Table 5 shows the mediating role of inflammatory levels in the association between unhealthy diets and depressive symptoms among Chinese adolescents. The direct effects of unhealthy diet scores on depressive symptoms were significant at all mediation models (*β* = 0.09–0.12, all *p* < 0.001). However, neither IL-4 nor CRP showed significant mediating effects at any time point (all indirect effects *p* > 0.05).

## 4. Discussion

Our study data showed that the prevalence of depressive symptoms among Yunnan adolescents was considerably high, and it was higher in females than in males. Moreover, regardless of sex, the prevalence of depressive symptoms demonstrated a clear upward trend from T1 to T5 across all groups. The prevalence of depressive symptoms among adolescents in our survey was comparable to that reported among adolescents in eastern China (36.2%) [68], India (34.9%) [69] and Bangladesh (36.6%) [70], but was generally higher than the prevalence reported among adolescents in the United Kingdom (24–32%) [71]. Adolescence is a critical stage of development in life, during which physiological, psychosocial, and cognitive changes predispose adolescents to mental health issues [3,72,73]. In recent years, the prevalence of depressive symptoms among adolescents has been prominent globally, with the highest prevalence of elevated depressive symptoms observed among adolescents in the Middle East, Africa, and Asia, and a higher prevalence among female adolescents [3]. Furthermore, a national survey in China revealed that depressive symptoms among adolescents exhibit distinct developmental trajectories. Females consistently exhibited higher levels of depressive symptoms between the ages of 14 and 18, whereas males demonstrated a less pronounced inverted U-shaped pattern, with a rebound observed in late adolescence [74]. Over the past three decades, unhealthy lifestyle behaviors have been prevalent among children and adolescents in China, while mental health issues have become increasingly prominent. However, relevant education, counseling, and treatment services have failed to keep pace with the growing demand [72]. Therefore, greater attention from the whole society should be directed toward the mental health of Chinese adolescents, with differentiated prevention and control measures implemented according to gender.

The present study found that unhealthy diets score associated with depressive symptoms across all groups: total sample, males and females. Unhealthy dietary behavior scores in adolescents from T3 to T5 exhibited autocorrelations, and depressive symptoms from T3 to T5 also exhibited autocorrelations. Unhealthy dietary behavior scores at T3 and T5 were associated with depressive symptoms at T3 and T5, respectively. Our study data support the findings of previous research. A study conducted in eastern China showed that, compared with adolescents who maintained healthy dietary behaviors, those with persistently relatively unhealthy dietary behaviors (*RR* = 1.80) and those with persistently very unhealthy dietary behaviors (*RR* = 2.45) were both at an increased risk of depressive symptoms [75]. Another study conducted in eastern China among adolescents aged 12 to 16 years found that the overall Global Dietary Recommendations score was negatively associated with depressive symptoms (*OR* = 0.92), while daily free sugar intake was positively associated with depressive symptoms [36]. A large-sample cross-sectional survey in China showed that a higher dietary diversity score was associated with lower depressive symptoms in adolescents, while the intake of SSBs, fried foods, fast food, and UPFs was positively associated with depressive symptoms [76]. Moreover, a systematic review and meta-analysis revealed that higher unhealthy dietary scores were positively associated with depressive symptoms in adolescents (*OR* = 1.20), whereas healthy dietary patterns were negatively associated with depressive symptoms (*OR* = 0.69) [77].

Numerous studies have demonstrated that diet plays a significant role in predicting mental health problems among adolescents. Among which, healthy dietary patterns are associated with fewer internalizing behavior problems, whereas unhealthy dietary patterns exacerbate such issues [78]. Healthy diets rich in complex carbohydrates, omega-3 fatty acids, B vitamins, and various amino acids are negatively associated with the incidence of depression, whereas unhealthy foods such as SSBs, UPFs, processed meats, sweets, snacks, and those high in saturated fatty acids are associated with an increased risk of depression in adolescents [31,33,79]. Specifically, a meta-analysis showed that unhealthy dietary patterns were positively associated with depressive symptoms in children and adolescents [34]. A survey conducted among 12 secondary schools in Chongqing, China, showed that compared with the group in the lowest tertile of SSBs intake, those in the highest tertile had a 62% increase in depressive symptoms over a four-year period [80]. Additionally, stratified analyses by sex suggest that a pattern characterized by high consumption of various healthy foods and low consumption of unhealthy foods and SSBs is significantly negatively associated with depressive symptoms in both male and female adolescents [81].

Adolescent depression is a major public health concern and has become a leading cause of disease burden among adolescents [82,83,84]. Diet, as a key modifiable factor, is increasingly recognized as important in the prevention and management of depression. Adjusting dietary patterns may represent a cost-effective and practical intervention strategy for the prevention and treatment of adolescent depression [31]. Consequently, cultivating a diverse and healthy dietary pattern may offer a promising and easily implementable approach [76]. Some scholars have proposed that reducing the intake of SSBs is a promising and implementable strategy for school-based health promotion and chronic disease prevention among adolescents in Asia [80]. A randomized controlled trial in Iran demonstrated that after a lifestyle intervention encompassing dietary behaviors, stress management, and physical activity, adolescents showed significant reductions in unhealthy dietary behaviors such as eating out, consumption of SSBs, and fast food intake, along with a significant decrease in depressive symptom scores [85]. Therefore, it is imperative to strengthen the development of health, disease prevention and control, and education systems, promote collaborative efforts across multiple sectors, while fostering supportive environments within families, communities, and schools, and actively engage children and adolescents in shaping their own healthy future [72]. For instance, an intervention study conducted in India demonstrated that a school-based behavioral intervention helped reduce adolescents’ intake of UPFs. Following the intervention, daily energy intake from UPFs decreased by 1062 kcal, and energy intake from processed foods decreased by 274 kcal [86]. Furthermore, community-level intervention strategies can also effectively improve adolescents’ dietary quality, increase fruit and vegetable intake, and reduce the consumption of SSBs [87]. It is noteworthy that adolescents’ dietary behaviors are influenced by upstream distal determinants, including the growth environment (e.g., community food environment, distribution of fast-food restaurants and convenience stores around schools), family economic status, and place of residence. If these structural factors are overlooked, dietary behavior interventions may only address symptoms rather than root causes, and fail to fundamentally prevent and improve depressive symptoms in adolescents. Therefore, constructing a multi-dimensional comprehensive intervention system to systematically optimize supportive environments for adolescent dietary health holds significant public health strategic importance.

Our study data showed that IL-4 and CRP at T3 were respectively associated with depressive symptoms in the total sample at T3, T4, and T5. IL-4 at T3 was associated with depressive symptoms in female adolescents at T3 and T4. IL-4 and CRP at T3 were associated with depressive symptoms in male adolescents at T5. Current evidence indicates that pro-inflammatory diets increase inflammatory levels, whereas anti-inflammatory diets can reduce them. Studies have found that adherence to a healthy dietary pattern (characterized by intake of whole grains, vegetables, fruits, micronutrients, and low sugar) in children and adolescents aged 2 to 19 years is associated with reduced levels of pro-inflammatory biomarkers (CRP, IL-6, and TNF-α); whereas adherence to an unhealthy dietary pattern (characterized by frequent intake of saturated fatty acids, UPFs, and sugary foods) is associated with elevated levels of these pro-inflammatory biomarkers [47,48]. The dietary inflammatory index (DII) is associated with elevated levels of various inflammatory markers in adolescents, including TNF-α, IL-1, IL-2, IFN-γ, and vascular cell adhesion molecule [43]. The Healthy Lifestyle in Europe by Nutrition in Adolescence (HELENA) study found that among adolescents aged 13–17 years, a diet rich in antioxidants and essential nutrients may alleviate the elevated levels of inflammatory biomarkers induced by obesity, whereas a poor-quality diet may predispose individuals to the early onset of oxidative stress [88]. A study from Brazil reported that among adolescents aged 18–19 years, higher scores of the energy-adjusted dietary inflammatory index were positively associated with higher levels of interferon-gamma [89]. Additionally, research data suggest that among European adolescents with the highest genetic risk for CRP, reductions in CRP levels were most pronounced in response to a healthier diet [90].

Epidemiological data indicate that pro-inflammatory diet is a significant predictor of depressive symptoms in adolescents. Multiple systematic reviews and meta-analyses have shown that, compared with anti-inflammatory diet, pro-inflammatory diet is associated with an increased risk of depressive symptoms (*OR* = 1.40). Anti-inflammatory diet may serve as an effective intervention strategy for reducing the risk of depressive symptoms [59,60].

A study from Iran reported that a pro-inflammatory diet was associated with severe (*β* = 1.67) and moderate (*β* = 3.96) depressive symptoms in adolescents aged 15–18 years [91]. Among Turkish adolescents, a higher DII was associated with depressive symptoms (*OR* = 2.90) [45]. However, the mechanistic link between dietary inflammation and depressive symptoms in adolescents remains unclear. On one hand, the impact of diet on chronic inflammation may stem from its antioxidant, anti-inflammatory, or pro-inflammatory properties, which may in turn give rise to alterations in the metabolism of inflammatory biomarkers [49]. Upon ingestion, unhealthy dietary components act as inflammatory inducers that are recognized by receptors expressed on immune cells, which serve as sensors for detecting such inducers. Subsequently, these sensors trigger the production of inflammatory mediators, including cytokines and chemokines [92]. Pro-inflammatory cytokines may contribute to depressive symptoms by reducing monoamine neurotransmitter levels in the brain, activating neuroendocrine responses, and promoting excitotoxicity [92,93,94]. On the other hand, chronic inflammation may damage brain plasticity and neural circuits, thereby affecting brain development and contributing to depressive symptoms in adolescents [93]. The polygenic score for C-reactive protein (PGS-CRP) serves as a tool to quantify an individual’s long-term genetic susceptibility to inflammation. Studies have found that genetic predisposition to inflammation influences cortical maturation and brain developmental trajectories by interfering with neurotransmitter systems, leading to cortical thinning, particularly in the right entorhinal cortex, right insula, and right superior temporal gyrus. Moreover, cortical thinning partially mediates the effect of PGS-CRP on depressive symptoms in adolescents [95].

The present study did not observe a mediating role of inflammatory levels in the association between unhealthy diets and depressive symptoms in adolescents. Limited research suggests that inflammation may mediate the association between diet and depressive symptoms in adolescents. For instance, the Western Australian Pregnancy Cohort (Raine) Study showed that unhealthy dietary patterns (characterized by high intake of red meat, fast food, refined foods, and confectionery) at age 14 were positively associated with inflammatory biomarkers at age 17, and such patterns increased the risk of depressive symptoms in adolescents through inflammatory pathways [51]. However, some studies have reported that the mediating role of inflammatory pathways in the association between dietary behaviors and depressive symptoms in adolescents is not significant. For instance, a study involving adolescents clinically diagnosed with depression showed that the association between inflammatory dietary patterns and depression weakened after adjusting for covariates [77]. A study conducted in Australia found that total dietary fiber intake was negatively associated with moderate-to-severe depressive symptoms in adolescents (*OR* = 0.27); however, the association was no longer significant after adjusting for inflammatory markers [40]. The Pelotas Birth Cohort Study showed that better diet quality was associated with a lower incidence of major depressive disorder in adolescence, with each one-standard-deviation increase in the diet quality score at age 18 being associated with lower CRP levels at age 18 and a reduced risk of major depressive disorder at age 22. However, no mediating effect of inflammatory markers was observed [96].

The failure to observe a mediating role of inflammatory levels in the association between unhealthy dietary patterns and depressive symptoms among adolescents in our study may be attributed to the following aspects. First, the dietary data in this study were collected through retrospective surveys, and the self-reported dietary behaviors of multi-ethnic adolescents in Yunnan may be subject to certain subjectivity and recall bias, which could have reduced the sensitivity to detect mediating effects. Second, the mechanisms underlying the influence of unhealthy dietary patterns on depressive symptoms in adolescents are complex and may involve multiple biological pathways. Unhealthy dietary patterns (characterized by the consumption of ultra-processed foods, exposure to endocrine-disrupting chemicals such as bisphenol A from food packaging, and high levels of fat, sugar, and salt) can induce gut microbiota dysbiosis, which subsequently disrupts central neuroendocrine homeostasis via the gut-brain axis [97]. Such dietary practices reduce the intake of neuroprotective nutrients, including eicosapentaenoic acid, docosahexaenoic acid (DHA), vitamin D, polyunsaturated fatty acids, and dietary fiber, thereby compromising endogenous neuroprotective mechanisms [98]. Moreover, this dietary pattern contributes to dysregulation of the hypothalamic-pituitary-adrenal axis; the resultant hypercortisolemia inflicts damage upon hippocampal neurons and impairs emotion-regulating circuits. In parallel, the high-fat and high-sugar components provoke oxidative stress, leading to an accumulation of reactive oxygen species that surpasses the antioxidant capacity, promotes neuronal membrane lipid peroxidation, and induces mitochondrial DNA damage [99]. Concurrently, these pathophysiological processes attenuate the emotion-regulatory functions of the prefrontal-limbic system, ultimately culminating in the onset of depressive symptoms in adolescents [100]. However, the present study only considered inflammatory pathways, and further in-depth investigation is warranted. Third, the mediating role of inflammatory levels in the association between unhealthy dietary behaviors and depressive symptoms in adolescents may be influenced by long-term cumulative effects and time-lag effects. Although this study followed participants over a two-year period, from a life-course perspective, the follow-up duration may still be too short for the biological effects to become evident.

This negative finding holds important value, as it suggests that the “diet-inflammation-depression” model derived from Western populations cannot be simply generalized to Chinese multi-ethnic adolescents. Instead, the association between diet and depressive symptoms in this population may be primarily mediated by non-inflammatory biological pathways or psychosocial factors. Therefore, future research will aim to construct a more culturally sensitive and population-specific model of adolescent depressive symptoms from the following two perspectives, thereby providing a foundation for precision interventions: First, multidimensional immunological indicators will be expanded to capture the real-time dynamic relationships among dietary behaviors, inflammatory fluctuations, and depressive emotions. Second, latent class analysis will be employed to identify distinct subtypes of dietary behaviors, exploring heterogeneous effects that may be masked by overall averages.

Strengths. First, it is the first to investigate the mechanism underlying the role of inflammatory levels in the association between dietary behaviors and depressive symptoms among multi-ethnic adolescents in Yunnan, thereby filling a gap in the relevant research in this population. Second, this study employed a longitudinal design to explore the longitudinal associations between more than 20 types of unhealthy dietary behaviors and depressive symptoms among multi-ethnic adolescents in China, thereby further enriching the research data pertaining to Chinese adolescents.

Limitations. Firstly, the use of retrospective questionnaire surveys to collect data on dietary behaviors and assess depressive symptoms among adolescents may introduce certain information bias. Secondly, although this study included more than 20 types of unhealthy dietary behaviors, it primarily focused on the frequency of these behaviors, with limited attention given to intake quantities, nutritional composition, and overall diet quality. Thirdly, although the study sample was recruited from a multi-ethnic adolescent population in Yunnan Province, China, some loss to follow-up occurred during the two-year follow-up period due to the COVID-19 pandemic and other factors, which may have affected the statistical power of the analysis. Fourthly, although a longitudinal design was employed in this study, which allows for the determination of temporal sequence between unhealthy dietary behaviors and depressive symptoms in adolescents, causality cannot be definitively established, as the association between dietary behaviors and depressive symptoms may be bidirectional.

## 5. Conclusions

In conclusion, our longitudinal study reveals a high prevalence and clustering of unhealthy dietary behaviors, including high consumption of sugar-sweetened beverages, ultra-processed foods (e.g., instant foods and snacks). These dietary patterns and elevated inflammatory markers independently contribute to the risk of depressive symptoms over time. Although inflammation did not mediate the association between unhealthy dietary patterns and depressive symptoms, this finding suggests that unhealthy diet may affect mental health through pathways beyond peripheral inflammation, such as gut microbiota dysbiosis or HPA axis dysfunction. Our results highlight the urgent need for school-based interventions targeting specific, modifiable dietary behaviors, which may offer a practical strategy for depression prevention and mental health promotion among adolescents.

## Figures and Tables

**Table 1:** The distribution of the prevalence rate of depressive symptoms in adolescents.

Variables	Category	Males					Females				
		Normal	Depressive Symptoms	Total	*χ* ^2^	*p*	Normal	Depressive Symptoms	Total	*χ* ^2^	*p*
Age(year)	12–13	1236	385	1621	23.07	0.000	1276	692	1968	10.02	0.007
	14	1117	482	1599			1054	707	1761		
	15–17	562	258	820			429	267	696		
Ethnicity	Han	1056	404	1460	26.25	0.001	1096	589	1685	16.75	0.033
	Hani	93	62	155			97	78	175		
	Yi	1182	433	1615			952	573	1525		
	Wa	61	19	80			46	24	70		
	Lahu	213	87	300			249	166	415		
	Bulang	36	29	65			69	46	115		
	Dai	121	49	170			129	91	220		
	Bai	117	33	150			72	58	130		
	Other	36	9	45			49	41	90		
Residence	Rural	2136	889	3025	14.25	0.000	2087	1278	3365	0.65	0.420
	Urban	779	236	1015			672	388	1060		
The only child in the family	Yes	618	247	865	0.28	0.600	476	264	740	1.48	0.225
	No	2297	878	3175			2283	1402	3685		
Family type	Two-parent family	2410	930	3340	1.78	0.619	2286	1329	3615	9.05	0.029
	One-parent family	304	111	415			239	176	415		
	Combined family	162	63	225			198	127	325		
	Other	39	21	60			36	34	70		
The number of close friends	0	20	51	71	273.28	0.000	20	41	61	184.12	0.000
	1–2	174	205	379			274	374	648		
	3–4	415	230	645			588	393	981		
	5–6	354	98	452			381	154	535		
	≥7	1952	541	2493			1496	704	2200		
Self-perceived socioeconomic status	Worse	58	57	115	71.03	0.000	72	58	130	54.98	0.000
	Poor	308	197	505			194	201	395		
	Medium	1826	649	2475			2008	1177	3185		
	Good	521	154	675			357	198	555		
	Better	202	68	270			128	32	160		
Father’s education level	Illiteracy	245	110	355	8.44	0.077	234	161	395	15.07	0.005
	Elementary school	702	273	975			661	439	1100		
	Secondary school	1408	497	1905			1268	772	2040		
	High school	387	178	565			380	205	585		
	University	173	67	240			216	89	305		
Mother’s education level	Illiteracy	321	144	465	8.09	0.088	306	199	505	9.81	0.044
	Elementary school	710	290	1000			716	449	1165		
	Secondary school	1417	493	1910			1235	775	2010		
	High school	284	121	405			314	151	465		
	University	183	77	260			188	92	280		
Father’s occupation	Farmer	1404	541	1945	8.08	0.152	1495	885	2380	35.50	0.000
	Civil servant	200	75	275			201	94	295		
	Worker	648	257	905			450	345	795		
	Merchant	283	82	365			263	112	375		
	Staff	91	39	130			75	75	150		
	Other	289	131	420			275	155	430		
Mother’s occupation	Farmer	1566	654	2220	18.19	0.003	1663	972	2635	26.04	0.000
	Civil servant	226	69	295			162	83	245		
	Worker	389	111	500			204	191	395		
	Merchant	326	109	435			273	142	415		
	Staff	102	48	150			140	70	210		
	Other	306	134	440			317	208	525		

**Table 2:** Association of unhealthy diets score with depressive symptoms in adolescents.

Model	Sample	Unhealthy Diets Score	*β*	SE	Wald *χ*^2^	*OR* (95%*CI*)	*p*
Model 1	Total	Q4	0.25	0.06	15.23	1.29 (1.13–1.46)	0.000
		Q3	0.01	0.07	0.01	1.01 (0.89–1.14)	0.914
		Q2	0.02	0.07	0.12	1.02 (0.90–1.17)	0.732
		Q1	0.00			1.00	
	Males	Q4	0.18	0.10	3.29	1.20 (0.99–1.46)	0.070
		Q3	−0.11	0.10	1.23	0.89 (0.73–1.09)	0.268
		Q2	−0.03	0.11	0.06	0.98 (0.79–1.20)	0.810
		Q1	0.00			1.00	
	Females	Q4	0.33	0.09	14.74	1.39 (1.18–1.65)	0.000
		Q3	0.11	0.09	1.52	1.11 (0.94–1.31)	0.218
		Q2	0.07	0.09	0.62	1.07 (0.90–1.28)	0.431
		Q1	0.00			1.00	
Model 2	Total	Q4	0.18	0.08	4.81	1.20 (1.02–1.41)	0.028
		Q3	−0.03	0.08	0.15	0.97 (0.82–1.14)	0.695
		Q2	0.00	0.09	0.00	1.00 (0.84–1.19)	0.982
		Q1	0.00			1.00	
	Males	Q4	0.18	0.13	1.93	1.19 (0.93–1.53)	0.165
		Q3	−0.13	0.13	0.97	0.88 (0.68–1.14)	0.324
		Q2	−0.07	0.14	0.26	0.93 (0.71–1.23)	0.612
		Q1	0.00			1.00	
	Females	Q4	0.22	0.11	4.12	1.25 (1.01–1.55)	0.042
		Q3	0.06	0.11	0.26	1.06 (0.86–1.31)	0.608
		Q2	0.06	0.12	0.26	1.06 (0.85–1.33)	0.613
		Q1	0.00			1.00	

Model 1: Unadjusted variables. Model 2: Adjusted for age, ethnicity, residence, the only child in the family, parental occupation, family type, parental educational level, the number of close friends, self-perceived academic stress, self-perceived socioeconomic status, family changes, hospital experience, smoking, alcohol consumption, family history of depression, left-behind experience, physical activity, the impact of COVID-19, symptoms of insomnia, and screen time. Also, the analysis of all participants also adjusted for sex.

**Table 3:** Association of inflammatory level with depressive symptoms in adolescents.

Depressive Symptoms	Adolescents	Variable	Model 1					Model 2				
			*β*	SE	95%*CI*	Wald *χ*^2^	*p*	*β*	SE	95%*CI*	Wald *χ*^2^	*p*
T3	Total	IL-4	−0.09	0.09	−0.27–0.08	1.07	0.301	−0.17	0.08	−0.32–−0.02	5.17	0.023
		CRP	0.11	0.37	−0.61–0.83	0.09	0.759	0.64	0.31	0.05–1.24	4.44	0.035
	Males	IL-4	0.13	0.13	−0.12–0.38	1.03	0.309	−0.08	0.11	−0.29–0.13	0.51	0.475
		CRP	−0.41	0.49	−1.37–0.55	0.70	0.403	0.34	0.41	−0.46–1.14	0.71	0.401
	Females	IL-4	−0.27	0.13	−0.52–−0.01	4.29	0.038	−0.22	0.10	−0.43–−0.02	4.50	0.034
		CRP	0.76	0.54	−0.30–1.83	1.98	0.159	0.76	0.44	−0.11–1.63	2.90	0.088
T4	Total	IL-4	−0.19	0.09	−0.37–−0.01	4.24	0.040	−0.26	0.08	−0.42–−0.09	9.54	0.002
		CRP	0.42	0.37	−0.31–1.14	1.27	0.261	0.87	0.34	0.21–1.53	6.70	0.010
	Males	IL-4	0.03	0.13	−0.22–0.27	0.04	0.834	−0.16	0.12	−0.39–0.07	1.80	0.179
		CRP	0.08	0.48	−0.87–1.03	0.03	0.876	0.75	0.44	−0.12–1.62	2.88	0.090
	Females	IL-4	−0.33	0.13	−0.59–−0.08	6.41	0.011	−0.25	0.12	−0.48–−0.02	4.57	0.033
		CRP	0.81	0.56	−0.28–1.90	2.12	0.145	0.59	0.50	−0.38–1.56	1.41	0.235
T5	Total	IL-4	−0.22	0.10	−0.41–−0.03	5.09	0.024	−0.26	0.09	−0.44–−0.09	8.49	0.004
		CRP	0.67	0.39	−0.10–1.43	2.93	0.087	1.07	0.36	0.37–1.78	8.89	0.003
	Males	IL-4	−0.09	0.14	−0.36–0.18	0.45	0.501	−0.30	0.13	−0.56–−0.05	5.36	0.021
		CRP	0.47	0.52	−0.56–1.49	0.79	0.375	1.18	0.49	0.21–2.14	5.71	0.017
	Females	IL-4	−0.30	0.13	−0.56–−0.04	5.10	0.024	−0.18	0.12	−0.42–0.06	2.10	0.147
		CRP	1.05	0.57	−0.06–2.16	3.41	0.065	0.73	0.52	−0.30–1.75	1.94	0.164

Model 1: Unadjusted variables. Model 2: Adjusted for age, ethnicity, residence, the only child in the family, parental occupation, family type, parental educational level, the number of close friends, self-perceived academic stress, self-perceived socioeconomic status, family changes, hospital experience, smoking, alcohol consumption, family history of depression, left-behind experience, physical activity, the impact of COVID-19, symptoms of insomnia, and screen time. Also, the analysis of all participants also adjusted for sex.

**Table 4:** Association among unhealthy diets, inflammatory levels, and depressive symptoms.

Path	*β*	SE	Est/SE	*p*
DS of T3 on UD of T3	0.122	0.028	4.326	0.000
DS of T3 on IL-4	−0.071	0.071	−1.012	0.312
DS of T3 on CRP	0.021	0.071	0.304	0.761
DS of T4 on UD of T4	0.021	0.023	0.913	0.361
DS of T4 on UD of T3	0.007	0.023	0.295	0.768
DS of T4 on IL-4	−0.094	0.048	−1.973	0.048
DS of T4 on CRP	0.067	0.047	1.418	0.156
DS of T4 on DS of T3	0.740	0.013	56.178	0.000
DS of T5 on UD of T5	0.055	0.023	2.367	0.018
DS of T5 on UD of T4	−0.032	0.024	−1.315	0.189
DS of T5 on UD of T3	−0.021	0.022	−0.948	0.343
DS of T5 on IL-4	−0.063	0.044	−1.431	0.152
DS of T5 on CRP	0.082	0.044	1.883	0.060
DS of T5 on DS of T4	0.583	0.024	23.814	0.000
DS of T5 on DS of T3	0.253	0.026	9.696	0.000
UD of T5 on UD of T4	0.477	0.024	19.739	0.000
UD of T5 on UD of T3	0.261	0.026	10.178	0.000
UD of T4 on UD of T3	0.564	0.020	28.815	0.000
IL-4 on UD of T3	−0.010	0.029	−0.339	0.734
CRP on UD of T3	−0.007	0.029	−0.244	0.807

DS: depressive symptoms; UD: unhealthy diets; T: time-point.

**Table 5:** Mediating role of inflammatory levels in the association between unhealthy diets and depressive symptoms among Chinese adolescents.

Mediator	Path	*β*	SE	Est/SE	*p*
CRP	DS of T3 on CRP	−0.04	0.03	−1.67	0.094
	DS of T3 on UD of T3	0.12	0.03	4.24	0.000
	CRP on UD of T3	−0.01	0.03	−0.25	0.799
	Indirect	0.00	0.00	0.21	0.832
CRP	DS of T4 on CRP	−0.05	0.03	−1.95	0.051
	DS of T4 on UD of T3	0.11	0.03	3.84	0.000
	CRP on UD of T3	−0.01	0.03	−0.25	0.799
	Indirect	0.00	0.00	0.23	0.822
CRP	DS of T5 on CRP	−0.01	0.03	−0.48	0.630
	DS of T5 on UD of T3	0.09	0.03	2.90	0.004
	CRP on UD of T3	−0.01	0.03	−0.25	0.799
	Indirect	0.00	0.00	0.10	0.918
IL-4	DS of T3 on IL-4	−0.05	0.03	−2.00	0.046
	DS of T3 on UD of T3	0.12	0.03	4.23	0.000
	IL-4 on UD of T3	−0.01	0.03	−0.37	0.715
	Indirect	0.00	0.00	0.32	0.750
IL-4	DS of T4 on IL-4	−0.07	0.03	−2.61	0.009
	DS of T4 on UD of T3	0.11	0.03	3.83	0.000
	IL-4 on UD of T3	−0.01	0.03	−0.37	0.715
	Indirect	0.00	0.00	0.33	0.740
IL-4	DS of T5 on IL-4	−0.04	0.03	−1.29	0.199
	DS of T5 on UD of T3	0.09	0.03	2.89	0.004
	IL-4 on UD of T3	−0.01	0.03	−0.37	0.715
	Indirect	0.00	0.00	0.27	0.791

DS: depressive symptoms; UD: unhealthy diets; T: time-point.

## Data Availability

Data is available from the corresponding authors upon reasonable request.
